# Changing Trends in B-Cell Non-Hodgkin Lymphoma Treatment: The Role of Novel Monoclonal Antibodies in Clinical Practice

**DOI:** 10.3390/cancers15225397

**Published:** 2023-11-13

**Authors:** Rita Tavarozzi, Giulia Zacchi, Daniela Pietrasanta, Gioacchino Catania, Alessia Castellino, Federico Monaco, Carolina Gandolfo, Paolo Rivela, Antonella Sofia, Noemi Schiena, Claudia Bertassello, Giulia Limberti, Francesco Zallio, Manuela Zanni, Marco Ladetto

**Affiliations:** 1 Department of Translational Medicine, University of Eastern Piedmont, 28100 Novara, Italy; paolo.rivela@ospedale.al.it (P.R.); cbertassello@ospedale.al.it (C.B.);; 2 Division of Hematology, Azienda Ospedaliera SS Antonio e Biagio e Cesare Arrigo, 15121 Alessandria, Italy; giulia.zacchi93@gmail.com (G.Z.); carolina.gandolfo.16@gmail.com (C.G.); noemi.schiena@ospedale.al.it (N.S.);; 3 Department of Hematology, Santa Croce e Carle Hospital, 12100 Cuneo, Italy

**Keywords:** B-cell non-Hodgkin lymphoma, novel monoclonal antibodies, novel immunotherapies

## Abstract

**Simple Summary:**

We are entering an exciting new phase in the history of non-Hodgkin lymphoma treatment. Advances in monoclonal antibody-based therapies, which began with rituximab in the 1990s but have only recently started to show their full potential, along with the development of cellular therapies using chimeric antigen receptor constructs, are changing the way we tackle these cancers. Indeed, several of these non-chemotherapeutic agents, even when used as monotherapies, can confer robust and long-lasting remissions and, in some cases, even hold the promise of potential cures. This represents a truly remarkable opportunity, especially for patients who have undergone extensive prior treatments—a possibility that would have been inconceivable until just a short while ago.

**Abstract:**

We are currently witnessing a dramatic shift in our approach to the treatment of B-cell non-Hodgkin lymphoma (B-NHL). In the evolving clinical landscape, novel treatments for this clinically heterogeneous disease span a wide range of interventions, encompassing targeted agents, cell therapy approaches, and novel monoclonal antibodies (NMABs). Among these, the latter are likely to exert the most profound impact due to their distinctive high efficacy and versatile applicability. NMABs represent a heterogeneous group of agents, including naked antibodies, immunotoxins, and T-cell-engaging molecules. In recent times, several NMABs have either gained regulatory approval or are on the verge of introduction into clinical practice, addressing multiple therapeutic indications and treatment regimens. Their anticipated impact is expected to be broad, initially in the context of relapsed/refractory (R/R) disease and subsequently extending to early treatment lines. The scope of this review is to provide a comprehensive overview of the biological characteristics, clinical properties, efficacy, and toxicity profiles of NMABs that have recently been introduced or are nearing integration into clinical practice.

## 1. Introduction

The anti-CD20 monoclonal antibody (MAB) rituximab initiated the era of cancer immunochemotherapy more than two decades ago, changing the therapeutic approach for B-cell non-Hodgkin lymphomas (B-NHLs). Since then, response rates and long-term disease-free survival have improved significantly across all B-cell lymphoma subtypes. However, a subset of patients (pts) with recurrent or relapsed (R/R) disease have proven more challenging to treat, showing lower responses to salvage therapies [1].

Novel monoclonal antibodies (NMABs) are a heterogeneous group of anticancer agents, as they include naked antibodies, immunotoxins, and T-cell-engaging molecules [2,3,4,5,6,7,8,9,10]. These innovative therapies are expected to have a broad impact on the treatment of various malignancies, particularly B-NHL. These agents have already found or will soon find applications spanning from treating relapsed disease to becoming first-line treatments, whether used as single agents or in combination with other anticancer drugs or biological agents.

Several NMABs have recently been approved or are about to be introduced into clinical practice for different therapeutic indications and in different treatment schedules. These include the antibody–drug conjugates polatuzumab vedotin (PV) [2,4], loncastuximab tesirine (lonca) [6,7], the anti-CD19 naked antibody tafasitamab [5], and the bispecific antibodis (bsAbs) mosunetuzumab, glofitamab and epcoritamab [2,8,9,10]. Other bsAb, such as odronextamab has achieved promising milestones, showing durable responses in R/R settings, including some pts who had previously experienced disease progression after CAR-T cell therapy [10]. 

The following sections summarize the general properties of different NMABs, focusing on those drugs that are expected to have greater clinical relevance.

## 2. The Phylogenic Tree: From Murine Models to Novel Immunotherapies 

Therapeutic MABs are a group of molecules targeting one or more specific antigens. These molecules display high heterogeneity in terms of protein sequence, structure, and antigen binding affinity. 

Murine antibodies were the first generation of therapeutic MABs ever developed. However, due to their lack of human structural components, they were soon found to trigger a human anti-mouse response (HAMA), resulting in a marked reduction in their efficacy. To overcome these problems, genetic engineering approaches were developed with the production of antibodies structurally closer to humans, known as chimeric MABs [11,12,13]. 

Rituximab, a chimeric anti-CD20 antibody, was the first member of this class to be introduced into clinical practice, improving B-NHL prognosis. The success of rituximab spurred the development of novel antibodies driven by the dual objective of reducing immunogenicity and enhancing therapeutic effectiveness. As a result, new generations of anti-CD20 antibodies emerged, further increasing the number of treatment options available. The second generation of anti-CD20 MABs comprised fully humanized IgG1 antibodies, while the third generation consisted of both humanized and engineered MABs [13,14,15]. 

To improve MAB efficacy, immunotoxins were developed, giving rise to a new class of compounds known as antibody–drug conjugates (ADCs), which include MABs connected through a covalent linker to a small cytotoxic payload consisting of chemotherapeutic drugs, bacterial agents, plant protein toxins (defined as immunotoxins), or radiopharmaceutical agents. Once attached to the corresponding cancer-cell-surface antigen, the ADC is internalized, releasing the cytotoxic payload, ultimately leading to cell cycle arrest and apoptosis. At present, the FDA has approved three ADCs: BV, PV, and lonca [2], while further investigations are underway for other cytotoxic ADCs [2,4,6,7].

Among ADCs, radiopharmaceutical drugs that combine radioisotopes with anti-CD20 antibodies to enhance tumor cell killing, an approach referred to as radioimmunotherapy (RIT), have seen relatively limited use despite their robust clinical effectiveness, mostly due to the inherent complexity associated with their delivery and management. The most widely employed radioimmunoconjugate was 90Y-ibritumomab tiuxetan, which was employed in both the US and Europe for the treatment of R/R follicular lymphoma (FL) with promising results. Unfortunately, despite its demonstrated efficacy, 90Y-ibritumomab tiuxetan is no longer available due to discontinuation in production [16,17]. 

New RIT options are currently undergoing evaluation. Among them, 177Lu-lilotomab satetraxetan, an RIT designed to target a less commonly targeted antigen, CD37, has been investigated in preclinical models, showing remarkable efficacy in clinical trials (LYMRIT-37-01; NCT01796171), making it a potentially attractive agent [18].

Bispecific antibodies (bsAbs) are antibodies, or parts of them, that can bind two different antigens (Ags), simultaneously engaging both tumor and immune effector T cells.

In the past, initial attempts were made to demonstrate that Abs capable of binding to two different domains could be combined, leading to enhanced activities. However, it was only through the improvement in their chemical structures that researchers succeeded in making them suitable for clinical investigation. 

Currently, there are two distinct antibody formats available: single-chain fragment variable (scFv)-based Abs, which lack a fragment crystallizable (Fc) region and are often referred to as “non-IgG-like” bsAbs, and full-length IgG molecules, known as “IgG-like” bsAbs. Several non-IgG-like bsAbs are being tested in human trials with different formats, such as bispecific T- or killer-cell engagers (BiTEs or BiKEs), dual-affinity re-targeting antibodies (DARTs), and tandem diabodies (TandAbs). In contrast, most IgG-like bsAbs are in use or about to be utilized for the treatment of B-NHL (e.g., mosunetuzumab, odronextamab, and epcoritamab) [19].

The field of bsAbs is highly dynamic and rapidly expanding, with over one hundred bsAbs currently being tested, extending their applications even beyond cancer treatment. Nevertheless, the most significant advances have taken place in a B-NHL setting, where they have emerged as viable treatment options, both as standalone therapies and in combination with other agents. 

**Figure 1:**
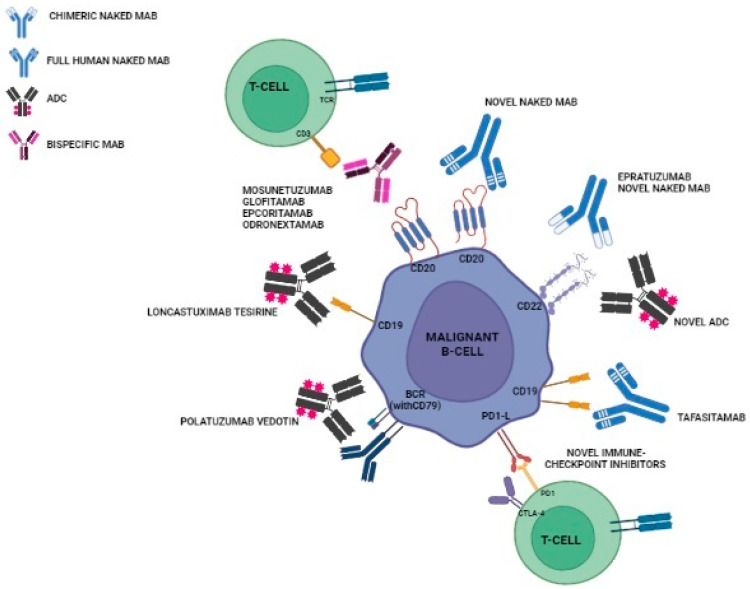
Relevant NMABs in clinical development.

Relevant NMABs in clinical development are represented in Figure 1. 

## 3. Relevant Novel MABs in Clinical Practice

### 3.1. Naked Antibodies

Rituximab has revolutionized the treatment course of CD20-positive B-NHL. Even though it remains the most administered MAB in the management of CD20-positive lymphomas, several NMABs have been developed to improve its effectiveness or overcome rituximab-refractory conditions [20,21,22,23]. These approaches involve the use of chemically modified anti-CD20 MABs or the investigation of alternative target antigens.

New generations of anti-CD20, either humanized or fully human, have been developed. Ofatumumab, a second-generation anti-CD20 MAB, has been approved for the treatment of R/R CLL, but the efficacy of ofatumumab in B-NHL pts has been far from convincing [14]. Third-generation anti-CD20 MABs are a group of fully humanized and engineered antibodies, which include obinutuzumab, ocaratuzumab, and PRO1319216. Among them, only obinutuzumab (GA101) has so far been approved by the FDA and European Medicine Agency (EMA) for the treatment of CLL and FL. In vitro studies have shown that obinutuzumab leads to more potent direct cell death (DCD) and antibody-dependent cellular cytotoxicity (ADCC) compared with rituximab. Several clinical trials have investigated whether obinutuzumab is superior to rituximab in the treatment of B-NHL pts. Unfortunately, the results were negative in aggressive lymphomas, while a modest, yet clear, effect was observed in patients suffering from indolent disorders [20,21,22].

Although anti-CD20 still remains one of the most important targets in B-NHL therapy, the search for novel targets has led to the development of several attractive NMABs. Among them, the most established one is the anti-CD19 antibody tafasitamab, which obtained accelerated approval from the FDA and EMA in 2020. 

#### Tafasitamab

The receptor for CD19 is an important functional regulator of normal and malignant B-cell proliferation and is expressed on all B-cell precursors. Notably, CD19 expression persists upon the downregulation of CD20 after rituximab exposure. Tafasitamab is the first-in-class, humanized monoclonal antibody engineered with an Fc region targeting CD19. Indeed, the engineering of FcγRIIa has led to not only enhanced binding to the stimulatory FcγRIIIa region but also reduced binding to inhibitory receptors, resulting in more potent ADCC [3]. 

In a phase II trial, tafasitamab monotherapy was administered to 92 pts with different R/R B-NHL types (i.e., DLBCL = 35; FL = 34; other indolent NHLs = 11; and MCL = 12). However, the outcomes were modest across all cohorts, with response rates of 26% in DLBCL, 29% in FL, and 27% in other indolent NHLs. Pts with rituximab-refractory disease showed a similar efficacy profile to pts with non-refractory disease. Tafasitamab tolerability was remarkably good, with infusion-related reactions (12%) and neutropenia (12%) reported as the most common side effects. No treatment-related deaths were reported, thus prompting the further investigation of tafasitamab in phase II/III combination therapy trials among R/R DLBCL pts [3]. 

Given its immunomodulatory properties, lenalidomide (LEN) emerged as the ideal companion for tafasitamab. This combination was evaluated in 80 R/R DLBCL pts ineligible for autologous stem cell transplantation (ASCT) [5]. The treatment regimen consisted of intravenous (IV) tafasitamab (12 mg/kg) and oral LEN (25 mg/day) administered for up to 12 cycles, each lasting 28 days. Subsequently, pts with stable disease or better continued with tafasitamab monotherapy until progressive disease (PD). With a median follow-up (mFU) of 13 months, all 80 pts achieved an objective response, resulting in an objective response rate (ORR) of 61%, including a complete response rate (CRR) of 43% and a 12-month progression-free survival (PFS) of 50% (95% CI: 38–61). Encouragingly, responses were seen across various risk categories, including cell of origin subtype and refractory status. The toxicity profile remained acceptable, with the most common adverse event being neutropenia (all grades), G1–2 diarrhea, and rash (32% and 27%, respectively) [5]. In additional follow-ups, the median duration of response (DOR) extended to 34.6 months, whereas the median overall survival (OS) reached 31.6 months, indicating response durability achieved through this immunologic chemo-free combination [24]. 

However, various real-world experiences were conducted with discordant results. The RE-MIND trial (NCT04150328) evaluated pts treated with tafasitamab + LEN monotherapy, confirming significantly better outcomes of this combination in ASCT-ineligible R/R DLBCL pts [25]. In contrast, a retrospective analysis of 82 R/R DLBCL cases treated with tafasitamab reported lower ORR, CRR, PFS, and OS compared to what was observed in the L-MIND trial. The authors attributed these differences to a greater incidence of high-risk conditions, increased rates of comorbidities, treatment delays, and dose reductions among real-world pts. Notably, pts with relapsed disease, low–moderate IPI scores (0–3), and fewer prior lines of therapy (LOT) (0–2) showed better outcomes, suggesting that individuals with lower-risk characteristics are the most suitable candidates for tafasitamab treatment [26]. In addition, a preliminary evaluation in a small series of R/R CAR-T pts showed potential benefits in this setting [27]. 

Overall, these findings underscore the complexities of applying tafasitamab to treat actual cases and highlight the importance of pt selection based on risk characteristics.

Considering these results, subsequent first-line trials were designed. The First-MIND (NCT04134936) trial is a phase Ib randomized study aimed to assess the safety and tolerability of R-CHOP + tafasitamab ± LEN in pts with previously untreated and newly diagnosed DLBCL and an International Prognostic Index (IPI) score of 2–5. As of the data cut-off (DOC) date (5 May 2022), 66 pts were randomized with an mFU of 17.6 months. The ORR and best response across all pts were notably higher in the experimental arm. This report showed that adding tafasitamab plus LEN to R-CHOP increased treatment efficacy compared to that of tafasitamab alone, resulting in durable responses in treatment-naïve DLBCL pts. The long-term safety profile of this combination showed no particular concerns [28], generating interest in further randomized studies. Furthermore, tafasitamab in combination with bendamustine compared to bendamustine plus rituximab is ongoing in R/R DLBCL pts who are ineligible for high-dose chemotherapy and ASCT (NCT02763319). The primary endpoint of this study, for which preliminary results are currently pending, is PFS, with a target enrollment of 450 pts. Finally, a phase III, open-label study of tafasitamab + R-CHOP ± LEN in pts with newly diagnosed DLBCL, aiming to enroll 880 pts, is currently being conducted, with the primary endpoint also being PFS (NCT04824092). 

In conclusion, tafasitamab plus LEN is an effective option for patients with R/R DLBCL who are not eligible for ASCT, and it serves as a bridge to ASCT and CAR-T therapy. In addition, tafasitamab itself shows promise in combination with other therapies. Nonetheless, further research is needed to identify CD19 masking phenomena during tafasitamab treatment, and it is crucial to conduct careful patient selection in light of real-life efficacy data. 

Overall, the optimal sequence for CD19-targeted therapies after R-CHOP has yet to be determined. The past few years have unveiled a novel landscape, including further ways to improve R-CHOP as a first-line choice. Tafasitamab, whether used alone or with LEN, seems to be an attractive therapeutic option for newly diagnosed DLBCL, with pending results from the phase 3 study. 

### 3.2. ADCs

ADCs are sophisticated molecules composed of an antibody linked to a cytotoxic drug. ADCs combine the targeting precision of MABs with the potent cancer-killing capabilities of drugs, allowing them to discern between healthy and diseased tissues [29].

In recent years, three ADCs, namely, brentuximab vedotin (BV) [30,31], PV [4], and lonca [6,7], have received approval from both the FDA and EMA, consolidating their role in the lymphoma treatment landscape. It is worth pointing out that BV, despite being the most established ADC, is primarily indicated for HL and T-cell NHL, limiting its use to these specific diseases. In addition, ongoing development efforts in this field are yielding several novel ADC molecules [32,33].

#### 3.2.1. Polatuzumab Vedotin 

CD79b is an essential component of the B-cell receptor (BCR) signaling pathway expressed on normal B cells and lymphomas [34]. PV is the first-in-class, humanized anti-CD79b monoclonal antibody linked to the cytotoxic drug monomethyl auristatin E (MMAE). In addition to MMAE-mediated cell death, PV can induce target cell death via antibody-mediated opsonization and antibody-dependent cell cytotoxicity [35]. 

PV was initially approved in combination with BR (bendamustine/rituximab) for R/R DLBCL pts after two or more lines of treatment who were ineligible for ASCT [4]. Approval was based on the results of a small, randomized phase Ib/II trial comparing PV in combination with BR vs. BR alone. This study enrolled 80 R/R DLBCL pts—40 in each arm (PV-BR vs. BR)—and showed improved rates of complete metabolic response (CMR), PFS, and OS in the experimental arm compared to the standard BR regimen. ORR and CR were 45% vs. 17.5% and 40% vs. 17.5%, respectively. The median OS was 12.4 vs. 4.7 months (*p* = 0.002). No substantial difference was reported across risk groups, albeit higher-grade (G > 3) toxicity, particularly neutropenia, was more prevalent in the PV-BR arm. Nevertheless, there was no excessive occurrence of infection-related adverse events (AEs) [4]. Furthermore, peripheral neuropathy occurred in 44% of pts (all grades), which was reversible in most cases. Updated results from this study, including an extension cohort of 106 pts, further corroborated the previous results and showed significant survival benefits over an extended follow-up period [36]. A safety analysis of all pts receiving PV + BR in the safety run-in (*n* = 151) revealed that 121 (80%) pts experienced G > 3 AEs, with 55.6% of them having serious AEs. Infections, febrile neutropenia, and pyrexia were the most prevalent, whereas four pts (3%) reported secondary malignancies [36].

Real-world data collection from the UK involving 133 pts, who were treated between June 2019 and October 2020 with PV-BR has recently been published. These pts were stratified according to their treatment approach: 40 underwent bridging to CAR T-cell therapy and 13 received re-induction therapy with planned SCT consolidation, while 78 were administered stand-alone treatment without planned CAR T-cell therapy or SCT [37]. With a median follow-up period of 8.2 months, the results revealed that the stand-alone cohort achieved an ORR of 65.8% with 39.7% of pts obtaining CR. The median PFS was 5.4 months (95% CI: 3.0–10.8 months), and the median OS was 10.2 months (95% CI: 5.2–14.3 months). The 12-month PFS rate was 37% (95% CI: 24–50%) with improved PFS for pts achieving CR. In contrast, pts with bulky disease (>7.5 cm), at least one previous treatment, and refractory disease showed inferior PFS. Notably, in the CAR T-cell bridging cohort a higher ORR of 42.1% was observed. Among those failing CAR T-cell therapy, an ORR of 43.8% and a CRR of 18.8% were reported [37].

While PV is approved for use in combination with bendamustine, some major considerations need to be raised. Bendamustine has a long wash-out time of at least 12 weeks, which may be problematic for patients who are scheduled for CAR-T therapy. However, PV may serve as a bridge to CAR-T treatment. To address this issue, researchers have explored the potential benefits of PV in combination with other immunotherapies in various studies. Specifically, in the phase II, randomized ROMULUS trial, pts were randomly assigned (1:1) to receive either R-PV or R-pinatuzumab (375 mg/m^2^ rituximab plus 2.4 mg/kg ADCs) every 21 days until PD or unacceptable toxicity for up to 1 year. The trial enrolled 81 pts with DLBCL and 42 with FL. The results showed an ORR of 26% in R-pinatuzumab-treated pts vs. 54% in the R-PV arm. Among the 21 FL pts who received R-pinatuzumab, 62% achieved ORR, while R-PV-treated pts obtained a 70% ORR. An overall benefit–risk favoring R-PV was also reported. Considering these results, PV was selected by the study funder for further development in B-NHL [33]. 

A primary analysis of a phase Ib/II study assessing the feasibility, efficacy, and safety of a triple combination of PV, obinutuzumab (G), and LEN (PV-G-LEN) in 56 pts with R/R FL [38] revealed an ORR of 76%, with a CR of 65%. Among pts with refractory disease, the CR rate was 71%. The most relevant G > 3 AEs were neutropenia (55%), thrombocytopenia (25%), infections (25%), and anemia (14%). Notably, significant rates of dose reduction (32%) or delays (77%) were reported, primarily attributed to LEN. A more extensive follow-up period is currently underway [38]. 

Considering these data, a large phase III, placebo-controlled, double-blind trial named POLARIX was conducted to investigate the value of a combination where PV replaced vincristine in a CHOP-like schedule (PV-R-CHP) as first-line treatment for stages II-IV DLBCL [39]. In this study, 879 pts were randomized 1:1 between PV-R-CHP and R-CHOP. After a median follow-up of 28 months, PFS was significantly higher in PV-R-CHP-treated pts (77%; 95% CI: 73–81) than in the R-CHOP group (70%; 95% CI: 66–75) at 2 years. Overall survival at 2 years did not differ significantly between the groups (89%; 95% CI: 86–92 months in the PV-R-CHP group and 88.6%; 95% CI: 86–92 months in the R-CHOP group). The safety profile was similar in the two groups [39]. Interestingly, low M1 macrophage levels were associated with lower PFS in the R-CHOP group but not in PV-R-CHP-treated pts, suggesting that PV may affect both the lymphoma microenvironment and the treatment outcome [40]. Even though a modest benefit in PFS was observed, the absence of a corresponding improvement in OS raised concerns, especially when considering the high cost of PV therapy, for which the use of PV in early settings must be balanced against the significant cost increase in the early phase. In particular, the lack of benefit of PV-RCHP in several subgroups of DLBCL patients, including those aged 60 or younger, GCB subtype, double or triple hit case, extensive disease, and IPI score ≤ 2, may potentially limit its application as a new first-line standard of care.

A retrospective analysis compared outcomes of pts treated with PV-R-CHP and R-CHOEP in the high-risk DLBCL setting. Both regimens gave excellent results, with approximately 85% of pts surviving after two years. The rates of cytopenia, infection, and sensory neuropathy were greater in R-CHOEP-treated pts than PV-R-CHP-treated pts [41].

Several ongoing phase I/II studies are evaluating the potential benefits of PV in various types of lymphomas, such as newly diagnosed double- or triple-hit lymphomas (NCT04479267) and other untreated aggressive B-cell lymphoma histotypes (NCT04231877). 

#### 3.2.2. Loncastuximab Tesirine 

Lonca (ADCT-402) is an ADC combining a humanized anti-CD19 MAB CD19 with a pyrrolobenzodiazepine (PBD) dimer cytotoxin [42]. PBD molecules are sequence-selective, non-distorting, and potent cytotoxic DNA crosslinking agents that lock DNA strands, disrupting all DNA metabolic processes [6]. Preclinically, lonca has shown highly targeted antitumor effects, with DNA-PBD crosslinks persisting for up to 36 h [42].

More recently, the FDA and EMA have granted accelerated approval to lonca for the treatment of pts with R/R DLBCL after two or more prior LOT. Indications include DLBCL not otherwise specified (NOS), DLBCL arising from low-grade lymphoma, and high-grade B-cell lymphoma. This approval was based on data from studies that included pts with adverse prognostic factors. 

In a large phase I study involving 183 highly pretreated R/R B-NHL pts, lonca monotherapy was administered in 21-day cycles until either PD or unacceptable toxicity occurred. These pts achieved an ORR and CR rate of 45.6% and 27%, respectively. When categorized through histology, the ORR was 42% for pts with DLBCL (137 evaluable), 47% for pts with MCL (15 evaluable), and a remarkable 79% among pts with FL (14 evaluable). The median DOR for all pts was 5.4 months (95% CI: 4.0 months to not reached). In particular, it was 4.5 months (95% CI: 4–10 months) in DLBCL pts and “not reached” in MCL or FL pts. The most frequently reported AEs were hematologic alongside fatigue [6]. 

The pivotal phase II multicenter, open-label, single-arm, LOTIS-2 clinical trial [7] aimed to assess the efficacy and safety of lonca in R/R DLBCL pts aged 18 years or older who had undergone two or more lines of prior therapy. A total of 145 pts were enrolled and received at least one dose of lonca, with 137 (94%) pts eventually discontinuing treatment, primarily due to PD (81 pts, 59%). Of the 145 pts, 70 achieved either CR (*n* = 35) or PR (*n* = 35), resulting in an ORR of 48.3% (95% CI: 39.9–56.7). The median DOR was 10.3 months with a median time to response of 1.3 months. The most common G > 3 AEs were neutropenia (37 pts, 26%), thrombocytopenia (26 pts, 18%), and increased gamma-glutamyltransferase (24 pts, 17%). Treatment-emergent adverse events (TEAEs) with a fatal outcome occurred in 8 (6%) of 145 pts; none were considered related to the experimental drug [7]. 

With these encouraging results, lonca has shown potent and specific anti-tumor activity in lymphoma, both when used as a single agent and in combination with other approved drugs. These findings underscored the role of lonca as a promising agent in the B-cell lymphoma setting, thereby encouraging the development of novel clinical trials in this field. 

Congruently, a phase Ib, multicenter, open-label, multi-arm study is currently underway to evaluate the safety and anti-cancer activity of lonca in combination with gemcitabine, LEN, PV, or umbralisib in R/R B-NHL pts. This study aims to enroll approximately 200 participants and is still actively recruiting (LOTIS-7, NCT04970901). Furthermore, a phase III randomized study is ongoing, which compares lonca combined with rituximab vs. immunochemotherapy in R/R DLBCL pts (LOTIS-5, NCT04384484). The target enrollment for this study is 350 pts, and the primary endpoint is PFS. Moreover, a phase II study is assessing the efficacy of lonca as a consolidation strategy in DLBCL pts who have achieved PR after CAR T-cell therapy (NCT05464719). Finally, a multicenter, open-label, single-arm study is assessing the efficacy of lonca when administered as consolidation therapy following a short course of salvage immunochemotherapy in R/R MCL pts who have been treated with Bruton’s tyrosine kinase inhibitors (BTKis) or are intolerant to BTKi. The sponsor of this clinical trial is Fondazione Italiana Linfomi (FIL; NCT05249959). 

### 3.3. Bispecific Antibodies

Bispecific antibodies (bsAbs) are specifically designed to target molecules present on both tumor and T cells, triggering T-cell activation and immune-mediated cytotoxicity [43]. Importantly, all bsAb effects occur in an MHC-independent fashion, thus bypassing the restrictions imposed via MHC-T-cell receptor interaction. This feature is critically important as many B-NHLs, particularly DLBCL, frequently harbor genetic aberrations that result in the loss of MHC class I molecule expression [44].

BsAbs target a variety of cell-surface antigens, and they come in different formats. The FDA and EMA paved the way for bsAb R&D in 2014 with the approval of the first-in-class bsAb, blinatumomab, indicated for B-cell acute lymphoblastic leukemia (ALL), mainly due to its high efficacy in R/R ALL pts. However, when applied as a salvage strategy in B-cell lymphomas, blinatumomab only showed modest efficacy and limited feasibility, precluding further investigation in B-NHL trials. However, full-length bsAbs exhibited pharmacokinetic properties akin to those of MABs and endogenous IgG. This similarity allowed extending the dosing intervals, a key factor driving their development in the R/R B-NHL setting. 

Within this group, anti-CD20xCD3 bsAbs have shown remarkable single-agent activity in heavily pretreated B-NHL pts while maintaining a manageable toxicity profile. CD20xCD3 bsAbs possess one or more CD20-binding sites, each targeting tumors with distinct avidity and antigen-binding capacity [44]. In the following sections, we will focus on the characteristics of T-cell-engaging bsAbs that are currently in advanced clinical development for the treatment of B-NHL.

#### 3.3.1. Mosunetuzumab

Mosunetuzumab (M) is a fully humanized bispecific IgG1 antibody recognizing CD20 on tumor cells and CD3 on T cells. What sets M apart is its modified Fc fragment, lacking both FcγR and the complement binding site while retaining a single CD20 binding site. M has recently received approval from both the FDA and EMA for its use in B-NHL as a result of its impressive effectiveness. 

The clinical potential of M monotherapy in pts with B-NHL R/R—both aggressive and indolent forms, aNHL and iNHL, respectively—was initially assessed in a phase I/Ib study. The dosing regimen of M involved a stepwise escalation on days 1, 8, and 15 of cycle 1, followed by a fixed dose on day 1 of each subsequent cycle, with a maximum of 17 cycles, depending on tumor response. A total of 230 heavily pretreated pts were enrolled. Of them, 129 (77%) had aggressive NHL, including DLBCL, tfFL, and MCL, whereas 68 (23%) had indolent NHL, consisting of marginal zone lymphoma, FL, and SLL. Of note, 18% of these pts had received previous CAR T-cell therapy. 

Early efficacy assessments showed high response rates in both cohorts with ORR rates of 35% (CR = 19%) and 66% (CR = 48%) in aNHL and iNHL, respectively [43]. Among pts with a history of CAR T-cell therapy or previous treatment with anti-CD20 R/R, their ORRs were 36.8% (CR = 26%) and 56% (CR = 55%), respectively.

Across all pt groups, the observed responses were durable over time with a median DOR of 20.4 months (95% CI: 16-NE) in iNHL pts and 22.8 (95% CI: 7.6-NE) in the aNHL group [43]. Most AEs (53.7%) occurred during the first 21 days of treatment. Common any-grade AEs were neutropenia (28.4%), cytokine release syndrome (CRS) (27.4%), hypophosphatemia (23.4%), fatigue (22.8%), and diarrhea (21.8%). Interestingly, the CRS events were mostly reversible, predominantly graded as <2, and primarily occurred during the first cycle. Only three pts required tocilizumab, while one pt required vasopressors for CRS management. Neurologic AEs (NAEs) were observed in over 10% of pts, with common manifestations being headache (18%), insomnia (12%), and dizziness (10%). G ≥ 3 NAEs or serious NAEs were reported in 4% of pts [45]. These safety data were consistent with those reported previously in a heavily pre-treated R/R CAR-T population [43]. Among the observed AEs, one pt with chronic active Epstein–Barr virus infection died from hemophagocytic lymphohistiocytosis, which was considered treatment-related, while one pt each passed away from sepsis (treatment-unrelated), Candida sepsis (treatment-unrelated), and pneumonia (treatment-related).

An updated analysis with a median follow-up of 28.3 months showed durable responses in pts achieving CR at the end of treatment (EOT). A high proportion of pts remained event-free at the two-year mark. In exploratory analyses, a similar DOR benefit was observed, regardless of whether pts achieved an early or late CR [46].

In addition to the promising results of IV M, a subcutaneous (SC) formulation of the drug was also tested in a clinical trial (NCT02500407). The initial report from this study evaluated 23 highly pretreated pts with different histology reports, including 57% that were refractory to last prior therapy and 70% that were refractory to prior anti-CD20 immunotherapy. The outcomes were quite encouraging, with extremely high ORR and CR rates observed. Specifically, in the indolent NHL cohort, the OOR was 86%, with a CR rate of 29%, whereas in aggressive NHL pts, the OOR was 60% and the CR was 20% [47]. Of note, the SC formulation displayed a lower incidence of CRS likely due to a low absorption rate and high bioavailability (>75%), confirming the favorable toxicity profile of the SC vs. IV formulation. The most common (>20%) AEs related to SC M were CRS (*n* = 8; 35%), headache (*n* = 5; 22%; all G 1), and injection site reaction (*n* = 5; 22%; all G 1). All CRS events occurred during cycle 1, with most being G 1 (*n* = 6; 26%) or G 2 (*n* = 2; 9%), and all events resolved without the need for tocilizumab treatment or intensive treatment. Isolated cases of immune-effector-cell-associated neurotoxicity syndrome (ICANS) not associated with CRS occurred in nine pts (all G 1), with headache (22%) and tinnitus (9%) being the most common AEs [47]. 

Building upon these positive results, M was tested in several combination therapies, with preliminary data now available and summarized below. 

Its well-known immunomodulatory characteristics make LEN an attractive candidate for combination with bsAbs. In an ongoing phase Ib study, a preliminary analysis is underway to assess the safety and efficacy of M plus LEN in R/R FL pts who had previously received at least one LOT. At the DOC date, 27 heavily treated pts were enrolled, showing a remarkable OOR of 92% and an acceptable safety profile [48]. These promising preliminary findings have provided the basis for the initiation of a randomized phase III study comparing the efficacy of the M plus LEN combination with the standard of care, rituximab plus LEN (R2) (NCT04712097). The primary endpoint for this study is PFS. Recruitment for the phase III trial commenced in 2021, and we are eagerly awaiting the results. 

In the context of first-line treatment, M has been investigated both as a standalone therapy and in combination with chemotherapy, and recent preliminary data have shed light on its potential.

A phase I/II study, named GO40554 (NCT03677154), has been actively assessing the effectiveness of M in two distinct pt groups: elderly pts (80 years of age or older) or pts aged 60–79 years with untreated DLBCL who were deemed ineligible for R-CHOP chemotherapy. As of the DOC date, 40 pts, with a median age of 84 years, participated in this study. Of them, 32 (80%) had an International Prognostic Index (IPI) score ≥2. These pts received a median of 6 cycles, ranging from 1 to 13 cycles. Regarding safety, 87.5% of pts experienced at least one TAE, and 37.5% of them had G 3–4 AEs (eight M-related). Common (>10%) TEAEs were CRS (*n* = 9, 22.5%), abdominal pain (*n* = 7, 17.5%), rash (*n* = 5, 12.5%), and neutropenia (*n* = 5, 12.5%). No fatal events or G ≥ 3 neurologic AEs were recorded. In terms of efficacy, the ORR was encouraging at 67.7%, with a CR rate of 41.9%. It is important to mention that of the 13 pts with CR, 4 maintained durable responses lasting ≥12 months from therapy initiation [49].

Similar efficacy data were unveiled during the phase Ib/II GO40515 trial, in which M was also evaluated in early stage DLBCL pts eligible for CHOP chemotherapy. The study employed a unique dosing strategy for M in cycle 1, involving step-up doses to minimize CRS, with the full dose given on day 1 of subsequent cycles in combination with CHOP. A total of 43 pts participated in this trial, including 7 pts with R/R NHL and 36 pts with newly diagnosed DLBCL, and received M-CHOP. The outcomes were highly encouraging: among R/R NHL, the ORR was 86%, with a 71% CR. Remarkably, previously untreated DLBCL pts displayed an even more pronounced ORR of 96%, with an 85% CR rate. Safety wise, G ≥ 3 AEs occurred in 37 pts (86%), with 19 (44%) experiencing serious AEs. The most common AEs were of a hematological nature. Importantly, CRS was reported in only two pts (29%) with R/R NHL, and one of them received tocilizumab for management. Among the 53% of previously untreated DLBCL pts who experienced CRS events, only one received tocilizumab. All CRS events occurred in C1, were resolved without sequelae, and did not result in any discontinuation or delay in treatment. Lastly, no ICANS events were observed [50].

Finally, mosunetuzumab has shown deep and long-lasting remissions with favorable safety profiles when used as a monotherapy in highly pre-treated patients with R/R NHL. In addition, M appears to be a promising candidate for combination strategies and has been preliminarily tested in combination with fixed doses of LEN or chemotherapy, showing encouraging preliminary anti-lymphoma activity. Further studies are ongoing to assess the efficacy of M in early phases.

#### 3.3.2. Glofitamab

Glofitamab is another fully humanized IgG1-like bsAb with a unique 2:1 structure. Even though, similar to M, its Fc structure lacks FcγR and the complement binding site, glofitamab features two CD20-binding domains—derived from type II CD20 IgG1 glycoengineered obinutuzumab— which improves its affinity for CD20-positive tumor cells. In a preclinical study, glofitamab has recently shown its superior potency compared to that of M [51]. Glofitamab has recently received approval from both the FDA and EMA for its use in B-NHL.

In a phase I/Ib trial, glofitamab was used as a single agent for the treatment of R/R B-NHL. The trial consisted of giving anti-CD20 obinutuzumab before initiating glofitamab treatment to prevent CRS by both binding to surface lymphomatous CD20 and depleting peripheral B cells. Glofitamab was administered IV with an escalated dosing schedule, either every 14 or 21 days, for up to 12 cycles. This study evaluated 171 pts, including both aggressive and indolent NHL (grades 1–3A FL), with a median age of 64 (22–85) years. These pts had undergone a median of 3 (1–13) prior LOT, with 91% of them displaying refractory disease. A promising clinical activity was observed across all doses. Among pts with aggressive B-NHL, ORR and CR were 48.0% and 33%, respectively—41% and 29% in pts with DLBCL and 55% and 35% in pts with transformed FL. In the FL cohort, 71% of pts achieved an ORR with a CR rate of 48% [9]. The median DOR reached 10.8 months (95% CI: 3.8 months—NE) accompanied by a median PFS of 11.8 months (95% CI: 6.3–24.2 months). AEs were reported in 98% of pts. The most common AE was CRS, occurring in 86 of 171 (50.3%) pts (G3 or 4: 3.5%). The incidence of CRS increased with dose but significantly declined after the first administration. Symptoms of ICANS were uncommon and resolved in all cases. G ≥ 3 neutropenia occurred in 25% of pts. Infections and febrile neutropenia manifested in 52% and 3% of pts, respectively [9]. Additional long-term analysis confirmed the induction of high CR rates thanks to the fixed-duration monotherapy offered to heavily pretreated MCL pts, most of whom had prior BTKi therapy. CRS events were manageable and mostly low-grade [52].

The results of the pivotal phase II expansion results in R/R DLBCL pts have been recently unveiled. As of 10 October 2022, 154 pts had received at least one dose of study treatment and were, thus, included in the final evaluation. These pts had been subjected to a median of three prior therapies, with the number of prior treatments ranging from two to seven. Furthermore, 33% of them had received prior CAR T-cell therapy, and 85% were refractory to their most recent treatment regimen. With a median study duration of 20.1 months, ranging from 0 to 32 months, comparable CR rates were observed in pts both with and without prior CAR T-cell therapy (37% vs. 39%). The median duration of CR (DoCR) was 24.1 months (95% CI: 19.8 months—NE), and approximately 70% of these pts achieved CR in remission at the 18-month follow-up. The 18-month OS rate was 41% (95% CI: 32.1–49.3) [53].

Glofitamab monotherapy has revealed a favorable risk–benefit profile and the potential to address an unmet clinical need by offering an effective and less toxic treatment option for R/R B-cell lymphoma pts. When given with cycle 1 step-up dosing together with obinutuzumab, it effectively mitigated CRS and achieved durable CR with a manageable safety profile. Based on these results, glofitamab has been used as a first-line treatment approach. In this regard, a preliminary report from a phase Ib study examined the addition of glofitamab to R-CHOP therapy. To mitigate CRS risk, pts received R-CHOP in C1 for tumor debulking. Subsequently, IV glofitamab was administered during C2—on day (D) 8 at 2.5 mg and D 15 at 10 mg—and continued at the target dose of 30 mg from C3 onward, following 21-day cycles. The study enrolled 56 pts with a median age of 68 years (ranging from 21 to 84 years), with the vast majority (96.4%) suffering from Ann Arbor stage III/IV disease. After a median follow-up of 5.6 months, spanning from 5.1 to 10.3 months, CMMR was 76.1% and ORR was 93.5%. G ≥ 3 AEs occurred in 71% of pts, with 23.2% of these related to glofitamab. SAEs were reported in 18 (32.1%) pts, with glofitamab-related SAEs in five (8.9%) pts. There were three cases (5.4%) of G 5 AEs including COVID-19 pneumonia (*n* = 2) and rituximab-associated IRR (*n* = 1). AEs leading to dose modification or interruption of glofitamab occurred in 11 (19.6%) pts, which included cases of COVID-19 pneumonia (*n* = 3) and COVID-19 infection (*n* = 2). The median dose intensity was 100% for all R-CHOP components. There were no severe Gr 3–5 CRS events, and Gr 1–2 CRS was observed in six (10.7%) pts. All the CRS events occurred during C2–3, and they were all resolved. No glofitamab-related ICANS were reported. Neutropenia was seen in 27 (48.2%) pts (Gr ≥ 3 neutropenia: Gr 3, *n* = 6; Gr 4, *n* = 19), and serious infections were seen in 9 (16.1%) pts [54].

#### 3.3.3. Epcoritamab

Epcoritamab (GEN3013), a full-length human IgG1 bsAb recognizing CD3 and CD20, was generated through controlled Fab-arm exchange and further developed for SC administration [55]. To optimize its use, several mutations were introduced to silence the Fc domain. 

Phase 1 of the EPCORE NHL-1 study, which enrolled R/R B-NHL pts, adopted a dose-escalation approach. Specifically, patients received SC epcoritamab according to a step-up protocol, which included predefined priming doses given over a 2-week period, followed by full doses ranging from 0.0128 mg to 60 mg, depending on the specific cohort. This strategy aimed to mitigate the severity of CRS. Epcoritamab was administered in 28-day cycles until PD or unacceptable toxicity. The primary endpoint of phase 1 (dose-escalation part) was to determine the maximum tolerated dose (MTD) to be used in the following phase 2 of the study. 

In phase 2, 73 pts were enrolled, with 68 of them receiving the full dose of the drug. Of these, 46 (68%) had DLBCL, 12 (18%) had FL, 4 (6%) had MCL, and 3 (4%) had high-grade B-cell lymphoma. Importantly, all of them had either relapses or were refractory to previous treatments with anti-CD20 monoclonal antibodies. On average, these pts had undergone three previous LOT, and six of them (9%) had previously received CAR T-cell therapy. Pts with R/R DLBCL showed a remarkable increase in ORR across all doses, with a 91% ORR (range: 59–100 months) and 55% achieving CR (range: 23–83 months), particularly noticeable with the 60 mg schedule. In these pts, the median time until response was 1.4 months (interquartile range (IQR): 1.3–2.6 months) with a median time to reach CR of 2.7 months (1.3–2.8 months). High effectiveness was also shown in FL pts who received a dose ≥0.76 mg (ORR = 90%; CR = 50%). Even in the small subgroup of four MCL pts, responses were observed, notably in two pts with the blastoid variant of MCL [56].

As of the DOC date, the primary reason for discontinuing the study was PD, accounting for 46 (68%) out of 68 pts. Among the most frequently reported TAETs, pyrexia was prevalent in 47 (69%) pts, which was primarily associated with CRS in 40 (59%) of these subjects, while injection site reactions were documented in 32 (47%) pts. No cases of febrile neutropenia nor dose-limiting toxicities were reported [56].

Updated results from the large B-cell lymphoma (LBCL) expansion cohort, featuring a longer median follow-up of 20 months, have been recently presented, revealing an ORR of 63.1% and a CRR of 39.5%. The median OS was 18.5 months for LBCL pts and 19.4 months for DLBCL pts. The median DoCR in both pt populations was 20.8 months, and OS was not reached among complete responders in either group. Among the most common TEAEs of any G were CRS (51%), neutropenia (24%), pyrexia (24%), fatigue (23%), nausea (22%), and diarrhea (21%). Nine pts (6%) had G1–2 ICANS, and one pt suffered from G5 ICANS, albeit there appeared to be confounding factors in this particular case [57].

In a subgroup analysis, pts naïve to CAR T-cell therapy achieved a 69% ORR and a 42% CR vs. 54% ORR, while those with a history of R/R CAR T-cell therapy achieved a slightly lower ORR of 54% and a CR rate of 34%. After a median follow-up of 10.7 months, the estimated median DOR was 12 months, with pts who achieved CR not reaching this endpoint [57].

These results provided strong support for the ongoing phase 3 study examining the combination of epcoritamab with R-CHOP vs. the investigator’s choice of standard-of-care chemotherapy in first-line pts (NCT05578976). In addition to this initiative, the EPCORE NHL-2 trial, a multicenter, open-label phase 1b/2 study, aimed to evaluate the efficacy and safety of epcoritamab in combination with other treatment agents in B-NHL pts. In this study, a total of 111 FL pts were enrolled in arms 2a and 2b, comprising the following baseline characteristics: pts (58%) with a Follicular Lymphoma International Prognostic Index (FLIPI) of 3–5; pts (60%) with stage IV disease; and pts (57%) who had received a single line of prior treatment with alkylating agents (92%) or anthracyclines (63%), with two pts having received prior CAR T-cell therapy. In 101 efficacy-evaluable pts, the ORR was 97%, with a CMR observed in 86% of cases. The estimated 6-mPFS reached 93%, and high ORR/CMR rates remained consistent across high-risk subgroups, particularly in the progression of disease within 24 months (POD24) group, which displayed a 95% ORR and an 82% CMR [58]. These findings indicate that epcoritamab could potentially mitigate the negative impact of POD24. In this regard, the investigators are planning to examine a separate POD24 cohort. Lastly, epcoritamab plus R2 is currently under investigation in the phase 3 EPCORE FL-1 trial (NCT05409066).

Epcoritamab has recently been granted first approval in both the United States and Europe for the treatment of adult patients with R/R not otherwise specified DLBCL, including DLBCL arising from indolent lymphoma and high-grade B-cell lymphoma after ≥2 lines of systemic therapy. The clinical development of epcoritamab as monotherapy and in combination with standard-of-care agents for the treatment of mature B lymphomas is ongoing worldwide with very promising preliminary evidence.

#### 3.3.4. Odronextamab

In the NHL R/R setting, odronextamab (REGN1979) stands out as a first-in-class, fully human IgG4-based CD20/CD3 bsAb, characterized by a hinge-stabilized structure. Its evaluation took place in a phase I study, conducted across multiple centers, featuring both dose-escalation and dose-expansion approaches, known as the ELM-1 trial. In this study, odronextamab was administered according to a step-up dosing regimen over three weeks, followed by a fixed weekly dose regimen until week 12. Successively, maintenance dosing was implemented. This study enrolled 145 heavily pretreated pts, with 94 participating in the dose-escalation phase and 51 in the dose-expansion phase. The median age of the enrolled pts was 67 years, and 42 (29%) pts had previously undergone CAR T-cell therapy. Furthermore, 119 (82%) pts had developed resistance to their most recent LOT. At the DOC date, in FL pts receiving 5 mg doses, the cumulative ORR was 51% (ORR = 91%; CR = 72%), whereas all DLBCL pts receiving >80 mg doses achieved CR, with an ORR of 53%. In CAR-T-cell-treated DLBCL, the ORR was 33%, with 27% of pts displaying CR [59]. The most common G ≥ 3 AEs were anemia (36 [25%]), lymphopenia (28 [19%]), hypophosphatasemia (27 [19%]), neutropenia (27 [19%]), and thrombocytopenia (20 [14%]). Serious AEs occurred in 89 (61%) out of 145 pts, with the most frequent events being CRS (41 [28%]), pyrexia (11 [8%]), pneumonia (9 [6%]), and infusion-related reaction (6 [4%]). Four deaths were recorded and considered related to odronextamab, with causes including gastric perforation, lung infection, pneumonia, and tumor lysis syndrome. G 3 neurologic AEs were noted in three (2.3%) pts, but only one of these events required treatment discontinuation. There were no G 4 or higher neurologic AEs [59].

ELM-2 is a global, multicenter study that enrolled adult R/R B-NHL pts who had experienced relapse or refractory responses to at least two previous therapies. IV administration of odronextamab was carried out in 21-day cycles, with a weekly step-up dosing during C 1 to mitigate the risk of acute toxicity. Published preliminary analyses encompassed both aggressive and indolent cohorts. 

At the DOC date, a total of 131 FL and 121 DLBCL pts had been enrolled. Among the indolent population, the median age was 61 years, ranging from 22 to 84, with 53% being male. These pts had a median of three prior LOT, and 71% were refractory to their most recent treatment. Over a median follow-up duration of 22.4 months, an impressive ORR of 82% (99/121) and a CRR of 75% (91/121) were recorded, demonstrating substantial efficacy even in high-risk subgroups. Furthermore, the responses were proven to be durable, with a median DOR for complete responders of 20.5 months. Median PFS was 20.2 months (95% CI: 14.8–NE), and median OS had not been reached (95% CI: NE–NE). TEAEs occurred in all pts and were considered treatment-related in 118 (90%) cases. Treatment-related G 5 AEs were reported for 3 pts, comprising pneumonia, progressive multifocal leukoencephalopathy, and systemic mycosis, leading to treatment-related AE discontinuation in 10 pts. The most common TEAEs (>30%, all grades) were CRS (56%), neutropenia (40%), and pyrexia (31%). No ICANS were observed [60]. 

In the aggressive cohort, the authors reported a median age of 67 years (24–88), 60% males, 80% Ann Arbor stages III-IV, 58% IPI score ≥ 3, median prior LOT of 2 (range 2–8), and 56% primary refractoriness. ORR and CR rates were 53% (48/90) and 37% (33/90), respectively. Importantly, all CRs proved to be durable, with median CR duration not reaching (95% CI: 10.2 months—NE). TEAEs occurred in 117 (97%) pts and were considered treatment-related in 102 (84%) pts. The most common TEAEs (>30% of all grades) were CRS (53%), pyrexia (41%), and anemia (34%). ICANS were reported in only 2 pts (4%) following a step-up dosing review, and both were low-grade; ICANS occurred in 6% of pts on the 1/20 regimen. Treatment-related G 5 AEs occurred in two pts (2%), while treatment-related AEs led to discontinuation of odronextamab in eight pts (7%) [61].

Odronextamab has shown high and durable CRR among patients with R/R NHL in a pivotal phase 2 trial (Table 1 and Table 2). 

## 4. Novel Promising Agents in Clinical Development

Recent advances in protein engineering and manufacturing technologies have spurred the development of more effective and practical NMABs. While several novel naked MABs are under investigation, the results of these studies are still in the early stages, and future research will be required to fully understand their potential [62]. 

In this exciting backdrop, the discovery of novel cellular pathways has ignited renewed interest in the field of cancer therapy. One promising target appears to be ROR1, a receptor tyrosine kinase expressed on the surface of malignant B cells and in some solid tumors, such as carcinoma, sarcoma, and melanoma. NVG-111 is the first humanized bsAb under evaluation that has shown preliminary tumor-cell-killing activity in vitro [63]. Currently, an ongoing phase 1/2 study in R/R CLL and MCL pts is evaluating an escalating dose schedule given via continuous infusion over 21 days followed by a 7-day period during which pts are kept off the drug. As of July 2022, 10 subjects, with a median age of 60 years, had been enrolled in the study. ORR was observed in 66% of subjects and included two CRs. AEs were predominantly limited to week 1 of C1 and were all reversible. 

One of the most potent anticancer mechanisms involves the action of immune cells. 

Furthermore, there is evidence that bsAb therapy can increase immune checkpoint expression, which is considered a significant escape mechanism in this type of therapy. To overcome these inadequate T-cell responses, bsAbs may be combined with checkpoint inhibitors, chemotherapy, costimulatory molecules, or oncolytic viruses [64]. 

In this context, MCLA-145, a bispecific antibody targeting CD137 and PD-L1, operates by triggering PD-L1-mediated T-cell inhibition and simultaneously stimulating T-cell activation and expansion through CD137 agonism [65]. MCLA-145 is currently being evaluated in an open-label, single-agent, dose-escalation study. Expansion cohorts are included to confirm the dose, assess safety, and gather preliminary efficacy data in pts with advanced or metastatic malignancies (NCT03922204) [65].

## 5. Conclusions and Future Directions

We are at the beginning of an exciting phase in the evolution of non-Hodgkin lymphoma treatment. Since the introduction of anthracyclines in the late 1960s, we have made significant strides; yet, the prospect of a true cure has largely hinged on chemotherapy-based regimens, with allogeneic transplantation as a notable exception.

The monoclonal antibody revolution, which started with rituximab in the 1990s but is only now fully realizing its potential, together with the development of cellular therapies employing chimeric antigen receptor constructs, is fundamentally reshaping the therapeutic paradigm for these neoplasms. Several of these non-chemotherapeutic agents can elicit profound and prolonged remissions, potentially leading to cures when used as single agents, even in highly pretreated pts—an opportunity that would have been unthinkable until recently. Moreover, the prospect of developing countless rational combinations among biologics—including small molecules, which may not be curative on their own in most settings but might offer substantial synergistic potential—stands as one of the most attractive fields of investigation across various NHL subtypes. These hold promise for effective and well-tolerated approaches in nearly all clinical settings [66].

However, several questions remain about the optimal sequencing and usage of NMABs throughout the treatment process, particularly in the context of CAR T-cell therapies. In this regard, it is important to highlight that NMABs offer the advantage of swift administration as off-the-shelf treatment, which would not be possible with CAR T cells. 

In addition, older patients with R/R B-NHL or comorbidities may not be suitable candidates for CAR T-cell therapy. Nevertheless, many uncertainties persist regarding the application of these agents. Indeed, it is only through extensive integration into routine clinical practice that the oncology community will be able to develop a learning curve, fostering wider adoption of these drugs. Concerns also extend to the adequacy of T-cell collection, which can influence the effectiveness of bsAbs, and the potential for increased infection risk associated with the Abs.

To accelerate and steer this exciting process toward its highest achievements, all stakeholders involved in this transformative endeavor must address several critical aspects that still represent unmet needs for our pts. These include the following:

- Mitigating toxicity: Long-term toxicity, a major burden endured by patients during the chemotherapy and transplantation era, must be minimized. We need treatments that spare patients unnecessary suffering;
- Adopting and refining fixed-duration regimens: Despite the advancements, continuous anticancer treatment still has a negative impact on our pts’ quality of life (QoL). Thus, further investigations of fixed-duration regimens are essential to improve pts’ well-being and that of their families;
- Pursuing a cure (or at least a functional one): Most lymphomas can be substantially reduced in severity, making long-term disease control an achievable goal, not merely an aspiration;
- Achieving global accessibility: Currently, most NHL pts reside in low- to middle-income countries, where access to cutting-edge treatments is limited. Tackling this disparity should be a major priority in the years ahead.

Overall, the availability of improved drugs marks a significant step forward in the quest to cure lymphoma. However, this progress does not make this mission any easier. It is crucial for doctors, nurses, pharmaceutical companies, regulators, policymakers, and pts and their organizations to be aware of these historic opportunities and the associated challenges. Together, they should collaborate to ensure the rapid and comprehensive use of the novel opportunities that will become available in the coming years.

## Figures and Tables

**Table 1:** Summary of main published trials with novel monoclonal antibodies (NMABs) in non-Hodgkin’s lymphoma (NHL).

NMAB	Target	Trial Phase	N	Treatment Scheme	Setting	ORR/CR (%)	mDOR (Months)
**Naked MAB**							
Tafasitamab	CD19	II [2]	35	Monotherapy	RR DLBCL	26/-	30
	CD19	II [5]	80	Tafa-LEN	RR DLBCL	61/43	12
	CD19	III [28]	66	Tafa + R-CHOP+-LEN	Untreated DLBCL	83/75	-
**Antibody–Drug Conjugate**							
Polatuzumab vedotin	CD79b	Ib/II [4]	80	R-benda-PV	RR DLBL	45/40	-
	CD79b	Ib/II [27]	440	PV-R-CHP	Untreated DLBCL	88/77	-
	CD79b	Ib/II [38]	56	PV-G-LEN	RR FL	76/65	-
Loncastuximab tesirine	CD19	I [6]	183	Monotherapy	RR NHL	45.6/27	5.4
**Bispecific MAB**							
Mosunetuzumab	CD20/CD3	I/Ib [30]	130	Monotherapy	RR aNHL RR iNHL	35/19 66/48	22.8 20.4
	CD20/CD3	Ib/II [32]	43	M-CHOP	DLBCL untreated RR	96/85 86/71	-
Glofitamab	CD20-CD20/CD3	I/Ib [35]	171	Monotherapy	RR NHL	48/33	5.5
	CD20-CD20/CD3	II [36]	107	Monotherapy	RR DLBCL	50/35	-
Odronextamab	CD20/CD3	I [37]	145	Monotherapy	RR NHL RR FL RR DLBCL	51 91/72 53/53	-
Epcoritamab	CD20/CD3	I [38]	73 12 46	Monotherapy	RR NHL RR FL RR DLBCL	88/38 90/50 68/45	-
	CD20/CD3	II [40]	157	Monotherapy	RR DLBCL	63/39	12

NMAB: novel monoclonal antibody; ORR: overall response rate; CR: complete remission; mDOR: median duration of response; RR NHL: relapsed refractory non-Hodgkin’s lymphoma; RR DLBCL: relapsed refractory diffuse large B-cell lymphoma; RR FL: relapsed refractory follicular lymphoma; aNHL: aggressive non-Hodgkin’s lymphoma; iNHL: indolent non-Hodgkin’s lymphoma; R: rituximab; PV: Polatuzumab vedotin; Tafa-LEN: tafasitamab + LEN; R-benda-PV: rituximab–bendamustine–polatuzumab vedotin; PV-G-LEN: polatuzumab vedotin–obinutuzumab-LEN; PV-CHP: polatuzumab vedotin–cyclophosphamide–doxorubicin–prednisone; M-CHOP: mosunetuzumab + cyclophosphamide–doxorubicin–vincristine–prednisone.

**Table 2:** Summary of main ongoing clinical trials with novel monoclonal antibodies (NMABs) in non-Hodgkin’s lymphoma (NHL).

NMAB	Target	Trial Phase	ID	Treatment Scheme	Setting
**Naked MAB**					
	CD19	III	NCT05429268	Tafa + LEN	RR DLBCL
Tafasitamab	CD19	Ib/II	NCT04661007	Monotherapy Tafa + LEN Tafa + parsaclisib Tafa + RCHOP	RR NHL
	CD19	Ib/II	NCT05626322	Maplirpacept (PF-07901801) + tafa + LEN	RR DLBCL
	CD19	Ib/II	NCT05455697	Tafa + LEN + retifanlimab + CHOP	DLBCL (untreated)
	CD19	II	NCT05583071	HD-MTX-Tafa-LEN-R	Untreated PCNSL
	CD19	II	NCT05788289	Tafa + LEN	RR MCL
	CD19	II	NCT04646395	Tafa + acalabrutinib	RR MZL
	CD19	II	NCT04974216	Tafa + LEN + rituximab	80 y/o or Older DLBCL (untreated)
	CD19	II	NCT04978584	Tafa + LEN + rituximab + acalabrutinib + CHOP	GCB-DLBCL (untreated)
	CD19	I	NCT03930953	CC-99282 + Tafa	RR NHL
**Antibody–Drug Conjugate**					
Polatuzumab vedotin (PV)	CD79b	Ib/II	NCT03533283	Glofitamab and atezolizumab or PV	RR NHL
	CD79b	I	NCT02611323	Obinutuzumab + R + PV + venetoclax	RR FL RR DLBCL
	CD79b	I	NCT04739813	Venetoclax + ibrutinib + prednisone + obinutuzumab + LEN	RR NHL
	CD79b	I/II	NCT04491370	Autologous stem cell transplant followed by PV	RR NHL
	CD79b	II	NCT04659044	PV + venetoclax + R + hyaluronidase human	RR MCL RR FL RR DLBCL
	CD79b	I	NCT04790903	Venetoclax in combination with PV + R and CHP	DLBCL (untreated BCL-2 IHC)
	CD79b	II	NCT05800366	Glofitamab + PV-R-CHP	DLBCL (high risk)
	CD79b	I/II	NCT06040320	PV + R	PTLD
	CD79b	I	NCT04231877	PV and combination chemotherapy	LBCL (untreated)
	CD79b	II	NCT05798156	R in combination with glofitamab and PV	Untreated Aggressive LBCL
	CD79b	II	NCT04594798	PV, R and dose attenuated CHP in older patients	DLBCL
	CD79b	II	NCT05940051	ZPR regimen	RR DLBCL
	CD79b	II	NCT05940064	ZPR regimen in treatment-naïve elderly patients	DLBCL
	CD79b	II	NCT05169658	Mosunetuzumab with or without PV and obinutuzumab	RR NHL
	CD79b	II	NCT05410418	Mosunetuzumab and PV	Untreated FL
	CD79b	III	NCT04833114	PV + R + ICE (PV-R-ICE) vs. R-ICE alone	RR DLBCL
Loncastuximab tesirine (lonca)	CD19	II	NCT04970901	Lonca + gemcitabine + LEN + PV + umbralisib	RR NHL
	CD19	II	NCT05144009	Lonca + R	Untreated DLBCL (frail pts)
	CD19	III	NCT04384484	Lonca + R vs. R-Gem-Ox	RR DLBCL
	CD19	II	NCT05600686	Lonca + R-DA EPOCH	Untreated DLBCL
	CD19	I/II	NCT03684694	Lonca + ibrutinib	RR DLBCL RR MCL
	CD19	II	NCT05296070	Lonca	RR MZL
	CD19	II	NCT04998669	Lonca + R	RR FL
	CD19	II	NCT05249959	Lonca as consolidation after R-BAC	RR MCL
	CD19	II	NCT05222438	Lonca	High Risk DLBCL Post Transplant
	CD19	I	NCT05053659	Lonca+ venetoclax	RR DLBCL
**Bispecific MAB**					
Mosunetuzumab	CD20/CD3	I/II	NCT03671018	Mosunetuzumab + PV + R	RR NHL
	CD20/CD3	III	NCT05171647	Mosunetuzumab + PV vs. R-Gem-Ox	RR DLBCL and RR aggressive NHL
	CD20/CD3	I	NCT04246086	SC Mosunetuzumab + LEN vs. IV mosunetuzumab + LEN	RR FL
	CD20/CD3	I	NCT05464329	Mosunetuzumab in combination with platinum-based salvage chemotherapy	ASCT—eligible RR NHL
	CD20/CD3	II	NCT04792502	Mosunetuzumab with LEN augmentation	Untreated FL
	CD20/CD3	II	NCT06006117	Mosunetuzumab with LEN	RR MZL
	CD20/CD3	II	NCT05672251	Mosunetuzumab with lonca	RR NHL
	CD20/CD3	III	NCT04712097	Mosunetuzumab in combination with LEN vs. R in combination with LEN	RR FL
	CD20/CD3	II	NCT04889716	Mosunetuzumab (cohort 1) or obinutuzumab and glofitamab (cohort 2) when given after CAR-T cells	RR NHL
	CD20/CD3	II	NCT05260957	CAR-T cells followed by mosunetuzumab + PV	RR DLBCL and RR aggressive NHL
	CD20/CD3	II	NCT05169515	Mosunetuzumab or glofitamab in combination with CC-220 and CC-99282	NHL
	CD20/CD3	II	NCT05412290	Mosunetuzumab	Consolidation after autoSCT in R/R aNHL
	CD20/CD3	II	NCT05389293	Mosunetuzumab	Untreated FL
	CD20/CD3	II	NCT05169658	Mosunetuzumab monotherapy vs. mosunetuzumab + polatuzumab and obinotuzumab	RR indolent NHL
Glofitamab	CD20-CD20/CD3	Ib/II	NCT03533283	Glofitamab + atezolizumab or PV	RR NHL
	CD20-CD20/CD3	I	NCT03467373	Glofitamab + R or obinotuzumab or PV + CH(O)P	Untreated DLBCL or untreated NHL
	CD20-CD20/CD3	I	NCT05364424	Glofitamab in combination with R + ifosfamide, carboplatin, and etoposide phosphate	RR NHL
	CD20-CD20/CD3	I	NCT05219513	RO7443904 in combination with glofitamab	RR NHL
	CD20-CD20/CD3	I	NCT04077723	RO7227166 in combination with obinutuzumab or in combination with glofitamab following a pre-treatment dose of obinutuzumab	RR NHL
	CD20-CD20/CD3	I/II	NCT05533775	Glofitamab monotherapy and glofitamab + chemoimmunotherapy	RR NHL
	CD20-CD20/CD3	I/II	NCT05861050	Glofitamab with obinutuzumab, venetoclax, and LEN	RR MCL
	CD20-CD20/CD3	I/II	NCT05896163	Maplirpacept (PF-07901801) and glofitamab	RR DLBCL
	CD20-CD20/CD3	II	NCT04980222	Glofitamab in combination with R + CHOP	High-risk patients with untreated DLBCL
	CD20-CD20/CD3	II	NCT05800366	Glofitamab + polatuzumab-R-CHP	NHL
	CD20-CD20/CD3	II	NCT05798156	R-PV-glofitamab	Sixty-year-old patients ineligible for fully dosed R-CHOP DLBCL
	CD20-CD20/CD3	III	NCT04408638	Glofitamab in combination with gemcitabine + oxaliplatin vs. R in combination with gemcitabine + oxaliplatin	RR DLBCL
Odronextamab	CD20/CD3	II	NCT03888105	Odronextamab	RR NHL
	CD20/CD3	I	NCT05685173	Odronextamab	RR NHL
Epcoritamab	CD20/CD3	II	NCT05283720	Epcoritamab + LEN or ibrutinib or R-CHOP	RR DLBCL or untreated DLBCL
	CD20/CD3	I	NCT05206357	Epcoritamab	RR NHL
	CD20/CD3	II	NCT05660967	Epcoritamab with or without LEN	Untreated FL
	CD20/CD3	II	NCT05848765	Epcoritamab against standard chemotherapy	RR NHL
	CD20/CD3	II	NCT05852717	Epcoritamab with gemcitabine, dexamethasone, and cisplatin (GDP)	RR DLBCL
	CD20/CD3	III	NCT05409066	Epcoritamab + R + LEN	Untreated FL
	CD20/CD3	III	NCT05578976	Epcoritamab combined with intravenous and R-CHOP or R-CHOP	Untreated DLBCL
	CD20/CD3	II	NCT05783609	Epcoritamab + R	Untreated FL

NMAB: novel monoclonal antibody; ORR: overall response rate; CR: complete remission; mDOR: median duration of response; RR NHL: relapsed refractory non-Hodgkin’s lymphoma; RR DLBCL: relapsed refractory diffuse large B-cell lymphoma; RR FL: relapsed refractory follicular lymphoma; aNHL: aggressive non-Hodgkin’s lymphoma; iNHL: indolent non-Hodgkin’s lymphoma; R: rituximab; PV: polatuzumab vedotin; Tafa-LEN: tafasitamab + lenalidomide; R-benda-PV: rituximab–bendamustine–polatuzumab vedotin; PV-G-LEN: polatuzumab vedotin–obinutuzumab-LEN; PV-CHP: polatuzumab vedotin–cyclophosphamide-doxorubicin–prednisone; M-CHOP: mosunetuzumab plus cyclophosphamide–doxorubicin–vincristine–prednisone.

## Data Availability

Data are contained within the article.
